# Deep learning-based pectoralis muscle volume segmentation method from chest computed tomography image using sagittal range detection and axial slice-based segmentation

**DOI:** 10.1371/journal.pone.0290950

**Published:** 2023-09-05

**Authors:** Zepa Yang, Insung Choi, Juwhan Choi, Jongha Jung, Minyeong Ryu, Hwan Seok Yong

**Affiliations:** 1 Department of Radiology, Korea University Guro Hospital, Seoul, Republic of Korea; 2 Division of Pulmonary, Allergy, and Critical Care Medicine, Department of Internal Medicine, Korea University Guro Hospital, Seoul, Republic of Korea; Chung-Ang University Gwangmyeong Hospital, REPUBLIC OF KOREA

## Abstract

The pectoralis muscle is an important indicator of respiratory muscle function and has been linked to various parenchymal biomarkers, such as airflow limitation severity and diffusing capacity for carbon monoxide, which are widely used in diagnosing parenchymal diseases, including asthma and chronic obstructive pulmonary disease. Pectoralis muscle segmentation is a method for measuring muscle volume and mass for various applications. The segmentation method is based on deep-learning techniques that combine a muscle area detection model and a segmentation model. The training dataset for the detection model comprised multichannel images of patients, whereas the segmentation model was trained on 7,796 cases of the computed tomography (CT) image dataset of 1,841 patients. The dataset was expanded incrementally through an active learning process. The performance of the model was evaluated by comparing the segmentation results with manual annotations by radiologists and the volumetric differences between the CT image datasets of the same patients. The results indicated that the machine learning model is promising in segmenting the pectoralis major muscle, with good agreement between the automatic segmentation and manual annotations by radiologists. The training accuracy and loss values of the validation set were 0.9954 and 0.0725, respectively, and for segmentation, the loss value was 0.0579. This study shows the potential clinical usefulness of the machine learning model for pectoralis major muscle segmentation as a quantitative biomarker for various parenchymal and muscular diseases.

## Introduction

Computed tomography (CT) is widely used for diagnosis, patient evaluation, and monitoring of body composition changes. Volumetric techniques are essential for diagnostic purposes, and recent efforts focus on deriving non-invasive imaging biomarkers for various diseases [1, 2]. Hounsfield unit values enable assessment of the muscle, adipose tissue, and bone structures’ volume and status.

Chronic obstructive pulmonary disease (COPD) is a systemic disease with significant consequences, particularly in its relationship with sarcopenia [3–15]. As COPD progresses, it affects parenchymal function and the respiratory muscles, leading to decreased activity, reduced myogenic quality, oxygen utilization, and exercise capacity. Sarcopenia, characterized by diminished skeletal muscle mass, has been closely linked to COPD, as well as increased morbidity and mortality in various conditions, such as trauma and different cancers. CT plays a crucial role in diagnosing and monitoring sarcopenia by analyzing the changes in the muscle mass.

The pectoralis major is a crucial respiratory muscle covering a large part of the chest and connecting the shoulder and abdomen. Studies have demonstrated its volume and density to be closely associated with various pulmonary diseases [7, 9, 13, 15–26]. Respiratory muscle features are associated with parenchymal biomarkers, including airflow limitation severity and diffusing capacity for carbon monoxide [21, 22], used in diagnosing diseases, such as asthma and COPD. McDonald et al. found that the CT-derived pectoralis muscle area offers valuable indicators of COPD morbidity and is potentially more predictive than the body mass index [19]. Additionally, patients with high-density respiratory muscles who exercise may experience reduced symptoms associated with pulmonary disease, such as decreased shortness of breath [7, 23–25].

However, the body composition assessment was usually conducted manually by an experienced radiologist; the measurements of the muscle tended to be performed manually in specific slice locations of the CT volume, showing the correlation with the volume of interest. In-slice muscle area measurements may be distorted owing to muscle contraction, breath-holding, and patient positioning during the CT scanning process. The repeatability of the process might be assured by long-term education and experience; however, it is still time-consuming and expensive.

Previous studies of muscle segmentation on CT images have primarily relied on traditional image processing techniques, such as thresholding, region growing, and contouring, which may suffer from inaccuracies because of variations in image quality, contrast, and noise [27–29]. Some recent studies have attempted to employ machine learning and deep-learning approaches for muscle segmentation, showing promising results in terms of accuracy and automation [30–32].

González et al. proposed an efficient algorithm for segmenting the pectoralis muscle and adjacent structures [33], such as subcutaneous fat and the pectoralis minor, demonstrating performance within partially labeled datasets. However, as the primary goal was not automatic segmentation within a volume, the axial range was delimited. Meanwhile, recent deep learning-based approaches similar to that of our study have been reported [34–36], but these primarily focus on measuring the Pectoralis Major Area at specific locations, such as the aortic arch, in contrast to the comprehensive segmentation approach.

In this study, we aimed to address these limitations by developing a deep-learning-based automated volumetric pectoralis muscle segmentation method that can measure volumes and muscle mass for various applications. This comprehensive approach may be more useful in various clinical applications.

## Materials and methods

### Overall procedures

The proposed segmentation method utilizes deep-learning techniques to combine a muscle-area detection model and a segmentation model. The detection model was used to identify the vertical range of the pectoralis muscle from the patient’s CT volume dataset. Then, in the segmentation model, we segmented the actual pectoralis muscle area from the CT image slice.

**Fig 1:**
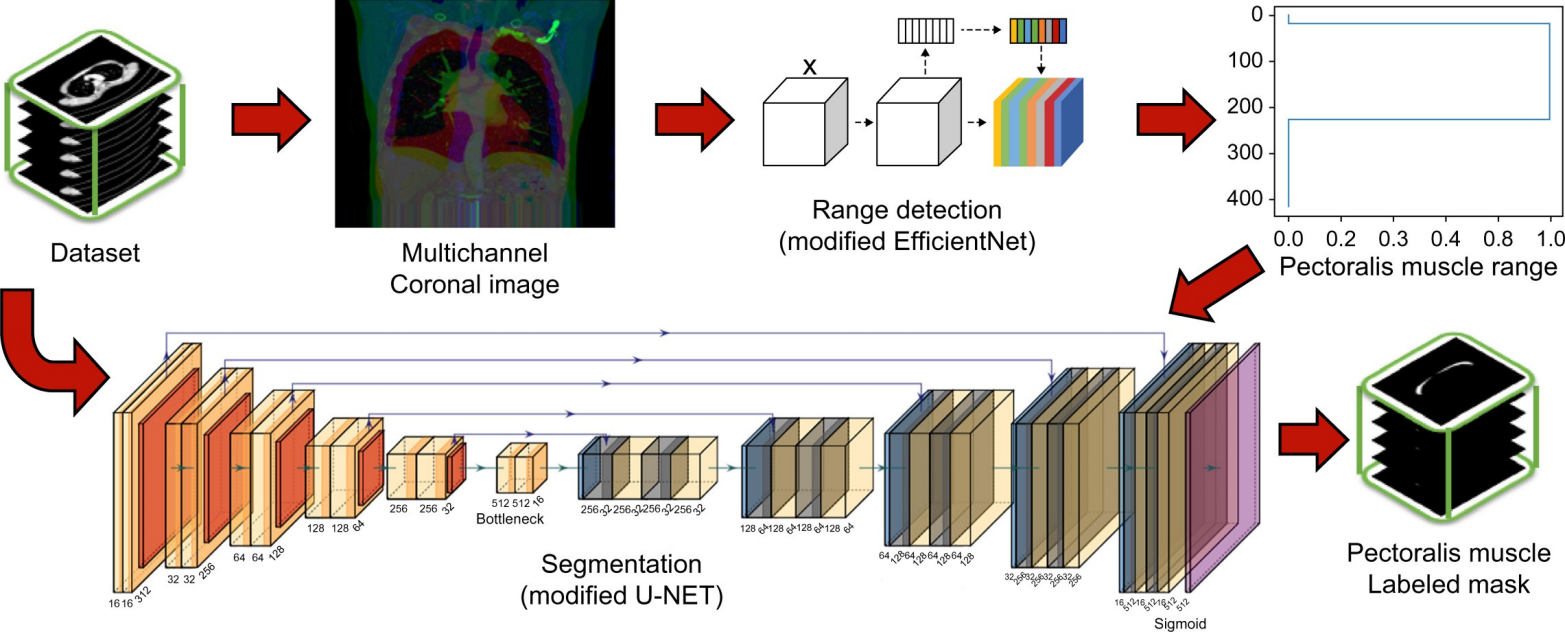
Overall process flow diagram of the proposed method.

The model was designed to separate the detection and segmentation stages to address the weight imbalance in the training dataset. It also allows for a more efficient deep-learning process and improves the performance of the voxel-based segmentation model. Fig 1 shows a diagram illustrating the overall process flow of the proposed method.

### Detection model

A binary classification model was designed to detect the vertical range of the pectoralis muscle from a patient image voxel dataset. The model was designed based on the modified EfficientNet (B6) model introduced by Tan et al. [37, 38]. As the compound scaling method was the primary concern of the model design, it showed promising results compared with other classification models with the dataset of the current study, with relatively fewer parameters and better efficiency.

A fully connected layer was added to the last layer to use the feature extraction function of the model. An adaptive average pooling method was used for the linearization of the intermediate result of the model because the length of the CT image dataset may vary owing to the scan conditions and parameters, which may lead to a nonlinear input data range. The training dropout ratio was set to 0.3 to avoid overfitting [39], and an additional linear function was adopted to determine the output feature size.

As the overall human organ structure information would be referred to in order to obtain vertical range detection, multiple coronal plane CT images with various locations were fused into multichannel images. With this method, we may lose the amount of image-based information, including the textures and precise volume sizes; however, it is relatively lightweight and produces accurate results because the model only aims to detect the vertical range.

For the training dataset, multichannel images of the patient combined with 2.5-dimensional sagittal information were analyzed. The labeled area masks in the coronal plane were set as the annotation data and used for the training dataset. The volume data of the CT image were interpolated into smaller volumes with isotropic voxel distance to avoid anisotropic transformation from the difference between the physical and voxel distances.

**Fig 2:**
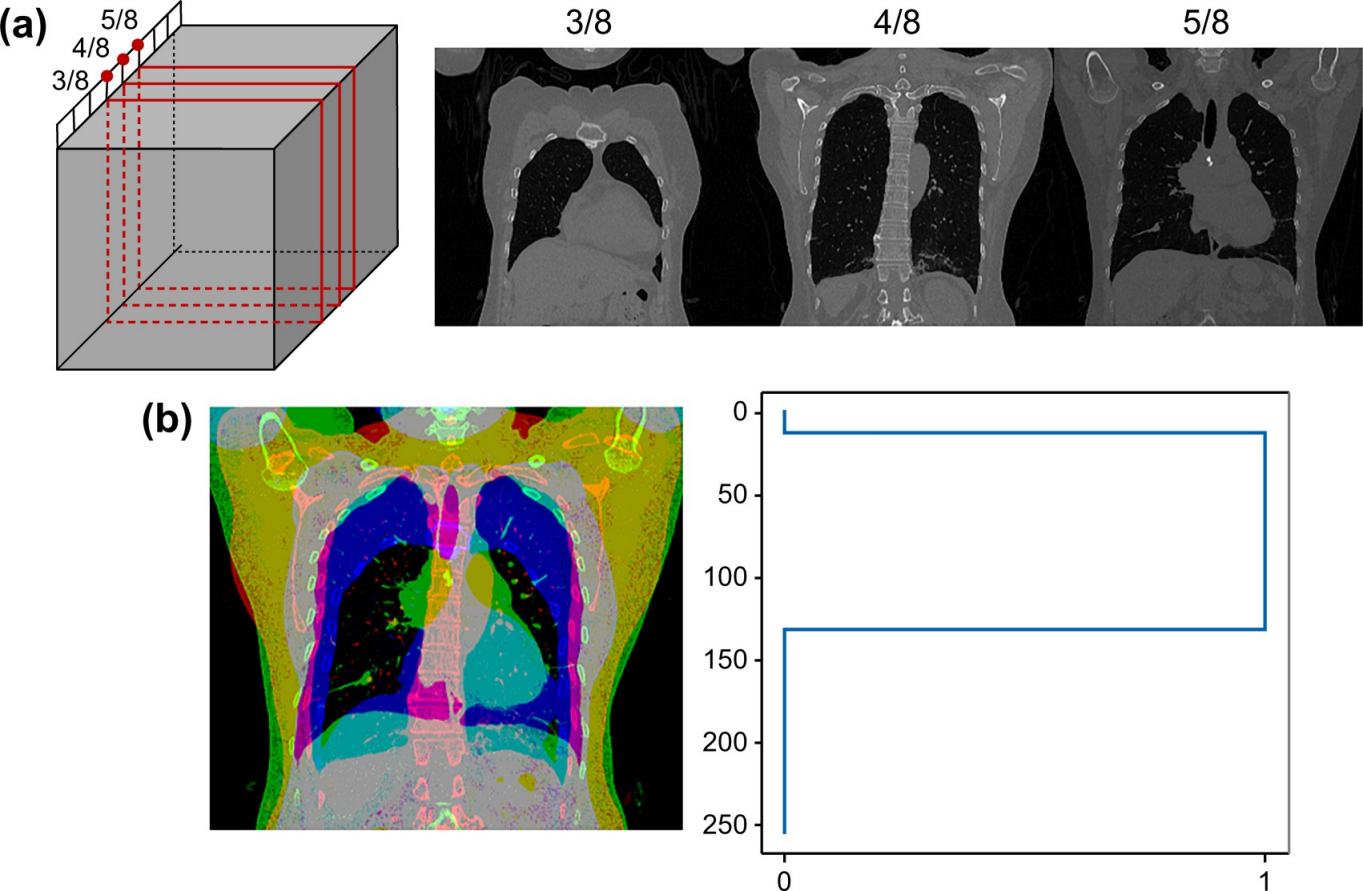
Multichannel coronal plane image generation method. (a) Three coronal two-dimensional images are acquired from the patient’s isotropic volume at the 3, 4, and 5 half-of-quarter positions, providing different perspectives of the pectoralis muscle area. (b) Fused multichannel image (left) and its labeled data (right) from the manually labeled pectoralis muscle area, demonstrating the combination of the three coronal images into a single multichannel image for input to the segmentation model and the corresponding ground-truth segmentation provided by expert radiologists.

From the patient CT volume image, three coronal two-dimensional (2D) images were acquired from 3, 4, and 5 half-of-quarter positions of the voxelized isotropic volume. Fig 2 shows the overall multichannel image fusion process and sample results.

In total, 10 images were acquired from a single patient by varying the acquiring position of the volume to augment the dataset and obtain the robustness of the model.

A binary cross-entropy loss function with logits was applied for the loss function as the range result was expected to be binary. Moreover, the outputs were thresholded to obtain binary predictions, which were then compared to the ground-truth labels. The accuracy was calculated as the proportion of correctly classified instances in each mini-batch, considering a threshold for binary predictions.

For prediction, the same procedure for acquiring multichannel coronal plane images was performed in an isotropic interpolated CT image volume. For better accuracy and vertical leakage prevention, five images were acquired by varying the acquiring position of the volume and input to predict the pectoralis major muscle. The predicted ranges of the five images were median calculated and used as the final range. Upon determining the vertical range of the pectoralis muscle presence, the corresponding slices were fed into the segmentation model to accurately delineate the axial area of the pectoralis muscle.

### Segmentation

**Fig 3:**
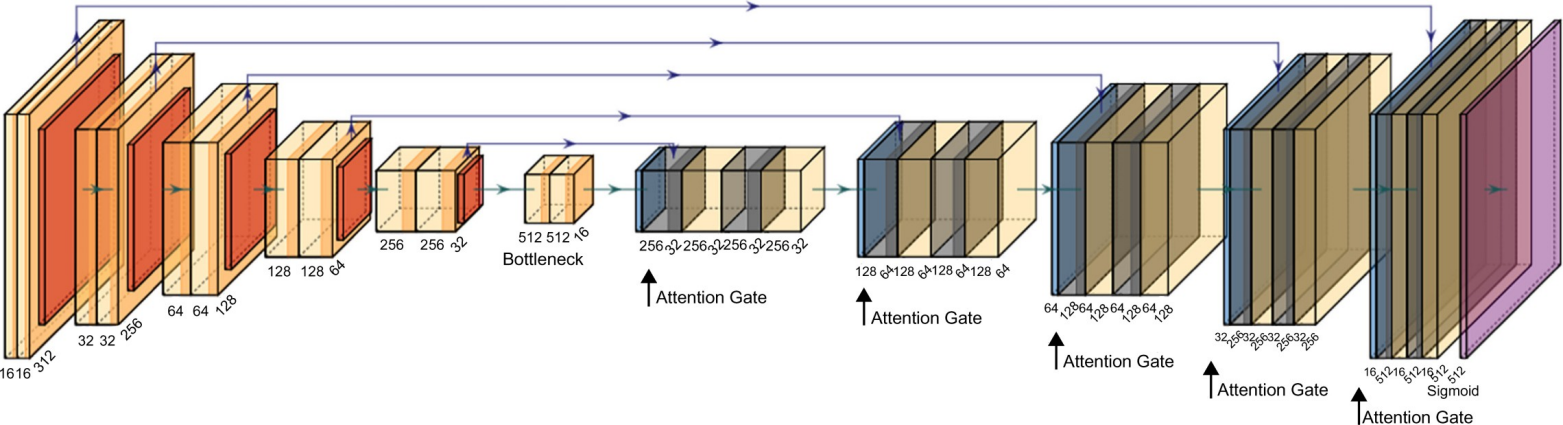
Graphical illustration of the proposed segmentation model based on the conventional 2D attention U-Net. An additional attention block is attached to each sampling process.

For the segmentation model, the conventional 2D Attention U-Net model [40] was modified with an additional fully connected layer and an attention block for additional feature attention in the upsampling and downsampling processes. Fig 3 shows a graphical illustration of the model. The Attention U-Net architecture was designed to increase the weight of the target area by adding an attention gate to the skip connection path, which provides spatial information in the U-Net model learning process.

The segmentation model comprised six layers in both the encoder and decoder paths, with two strides and two scale factors. Each layer consisted of a feature map with 16–512 channels. In every layer, two convolution operations were performed, maintaining the same number of channels, width, and height. Batch normalization was applied, followed by the Rectified Linear Unit as the activation function.

Several modifications were made to the conventional 2D U-Net model to better address the challenges of pectoralis muscle segmentation on CT images. Skip connections and attention blocks were added to the decoder path to provide spatial information during learning and apply soft attention on the areas being learned. The final layer was enhanced with an attention block for additional feature attention during the upsampling and downsampling processes.

For post-processing, a simple connected-component detection algorithm was employed to remove unnecessary disconnected small areas and prevent leakage, resulting in cleaner and more precise muscle boundaries. These modifications to the conventional 2D U-Net model were designed to address the specific challenges in pectoralis muscle segmentation from CT images and contribute to the improved performance of the proposed model.

The Dice loss function was employed to optimize the model, and accuracy was computed based on the proportion of correctly classified pixels, considering a threshold for binarizing the predicted segmentation.

Following the segmentation of the pectoralis muscle in each axial slice, a post-processing step was added to reduce the inter-slice gaps and enhance the overall segmentation contour quality. This step involves a series of morphological operations, including dilation and erosion, to smoothen the segmented boundaries and fill in any minor holes. Additionally, a 3D connectivity analysis is carried out to exclude any non-pectoralis major components that might have been misclassified during the segmentation process. Finally, inter-slice interpolation is used to ensure consistency and continuity between the segmented slices, thereby minimizing any discrepancies in the volume calculation. These post-processing steps are crucial to improve the segmentation’s overall robustness and accuracy, contributing to a more reliable quantitative analysis of the pectoralis muscle.

### Datasets: Active learning

The training dataset for the models was increased using an active learning process with multiple stages. This method improved the performance of the models while reducing the labeling burden and increasing time efficiency.

Over 32,000 slices of CT images from 178 patients were used for the initial training and validation datasets. The ground-truth labels for the pectoralis muscle area were manually segmented by three radiologists experienced in chest CT interpretation. The readers were blinded to the patient’s clinical information and independently delineated the pectoralis major muscle on each CT slice. In cases of disagreement between the two radiologists, a consensus was reached through discussion and consultation with a third senior radiologist to ensure the accuracy and reliability of the ground-truth labels.

The manual segmentation process involved identifying the pectoralis major muscle boundaries on axial CT slices and drawing contours around the muscle area. This process was repeated for all relevant CT slices. The final muscle volume was calculated by summing the area of the muscle across all slices and multiplying by the slice thickness. Minor flaws in the results of the pectoralis muscle volume mask label were edited and used for additional training to facilitate transfer learning.

The dataset was randomly divided into the training and validation datasets with a ratio of 8:2, and the data were augmented with random rotation, mirroring, elastic deformation, and scaling, among others. For better accuracy and robustness of the initial training model, the segmented results of additional cases of the CT image dataset from patients were generated using the initially trained model. These results were visually graded and modified by experienced radiologists and used as the training dataset for the next stage of the model. The overall characteristics of the patient dataset used in the active learning process are presented in Table 1.

**Table 1:** The overall characteristics of the patient dataset.

Characteristics	First dataset	Second dataset	Third dataset	Final dataset
Case count	202	448	2,337	7,796
Age (in years)	67.83 ± 10.88	70.01 ± 9.85	69.98 ± 10.44	69.69 ± 10.40
Male	153	125	1,733	5,762
Female	49	323	604	2,034
Tube voltage (kV)	120	120	120	120
Low-dose	‐	246	1,446	5,521
Normal dose	202	202	891	2,275
Slice thickness	3	3	3	3
COPD diagnosed	202	202	1,902	5,131
Normal participants	0	246	435	2,665

For the final training and validation of the model, 7,796 cases of patient CT images were obtained from the Korea University Guro Hospital. Of these, 5,131 cases were images of patients with COPD, while the remaining 2,665 cases were of normal individuals from the diagnostic cohort. All CT examinations were performed using a diagnostic routine with normal- and low-dose CT scans, and the CT images were reconstructed using a standard sharpness kernel with a simple iterative reconstruction method, which is the standard image reconstruction protocol for chest and lung imaging. The study was conducted in accordance with the Declaration of Helsinki and approved by the Institutional Ethics Committee of Korea University Guro Hospital (IRB No.: 2018GR0179 and 2021GR0335). The ethics committee waived the requirement for informed consent due to the retrospective nature of the study.

**Fig 4:**
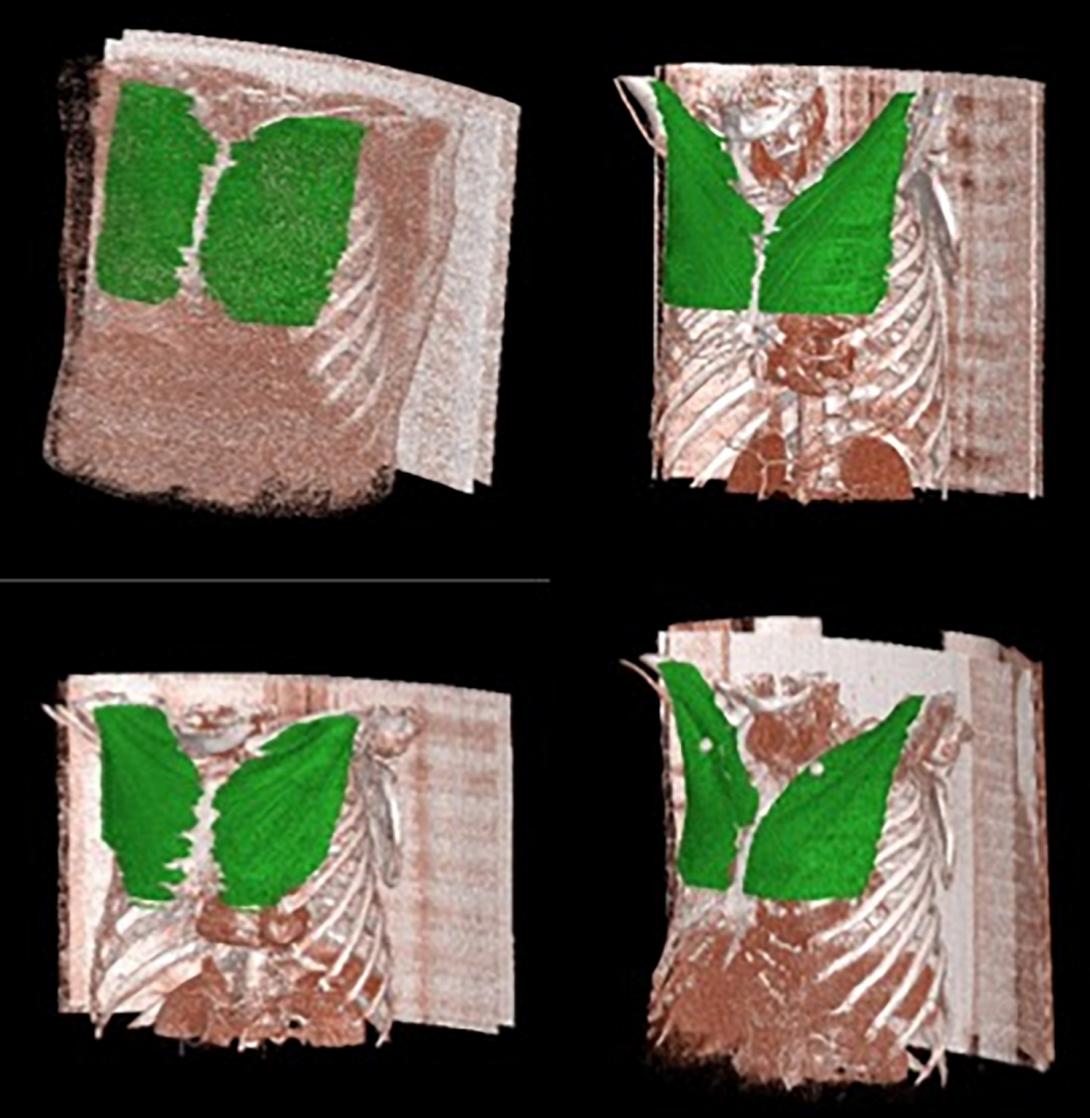
Sample case of a patient CT image with an overlayed label of the pectoralis major muscle.

The overall manual segmentation was performed using commercial image archiving and labeling software (AVIEW Metrics, Coreline Soft, Seoul, Korea) and an additional in-house-developed merging module. Fig 4 shows a sample case of a labeled patient CT image in the training dataset.

The noise content of CT images varies owing to many factors, such as imaging conditions, patient’s size, and patient’s shape. It could be assumed that a better segmentation result could be achieved if the standardization and leveling of the noise of the CT image preceded. Therefore, the noise reduction process for the overall CT image was performed using commercial image denoising software (ClariCT.AI, ClariPi, Seoul, Republic of Korea) as a preprocessing process.

### Training setup

The deep-learning models were designed using the PyTorch library (1.8.1) in acceptable hardware settings using an NVIDIA RTX2080Ti GPU. Training of the entire dataset was performed with a dual NVIDIA V100 GPU based on cloud-based hardware, provided by the National IT Industry Promotion Agency.

### Validation process during training and visual assessment

The results of the proposed method were evaluated using the Dice similarity coefficient (DSC) values, which could comparatively analyze the ground truth and predicted values. DSC was calculated by obtaining the ratio of the overlapped area between the predicted value and the ground truth divided by the sum of both areas. The DSC value was calculated as follows:

$$DSC\left(Vol_{result},\;Vol_{GT}\right)\;=\;\frac{2|Vol_{result}\cap Vol_{GT}|}{\left|Vol_{result}|+|Vol_{GT}\right|}$$

where $Vol_{result}$ is the segmented volume with the proposed method, and $Vol_{GT}$ is the volume labeled by experienced radiologists. The accuracy of the proposed segmentation method is higher when the value is closer to 1.

The segmentation results of the proposed method were visually assessed by experienced radiologists, technicians, and clinicians. The results were graded from 1 to 10 points based on accuracy, leakage, and under/over-segmentation.

### Evaluation of the method based on patient follow-up data

Patient chest CT follow-up data were used to evaluate the proposed segmentation method. For better accuracy of the overall process on the evaluation dataset, another 250 pairs of patients who did not have any particular findings related to the musculoskeletal system were included. The patients CT datasets were collected from Korea University Guro Hospital. The duration between the performed date of the two CT scans was less than 3 months. The CT dataset was processed using the proposed method, and the segmentation results were visually assessed by experienced radiologists to eliminate outliers and bad cases, such as a truncated muscle area, via selecting the region of interest (ROI) from the scanning process. The difference in the volume and density of the pectoralis muscle area was calculated by subtracting the labeled and predicted mask volumes and ROI in the CT dataset.

## Results

**Fig 5:**
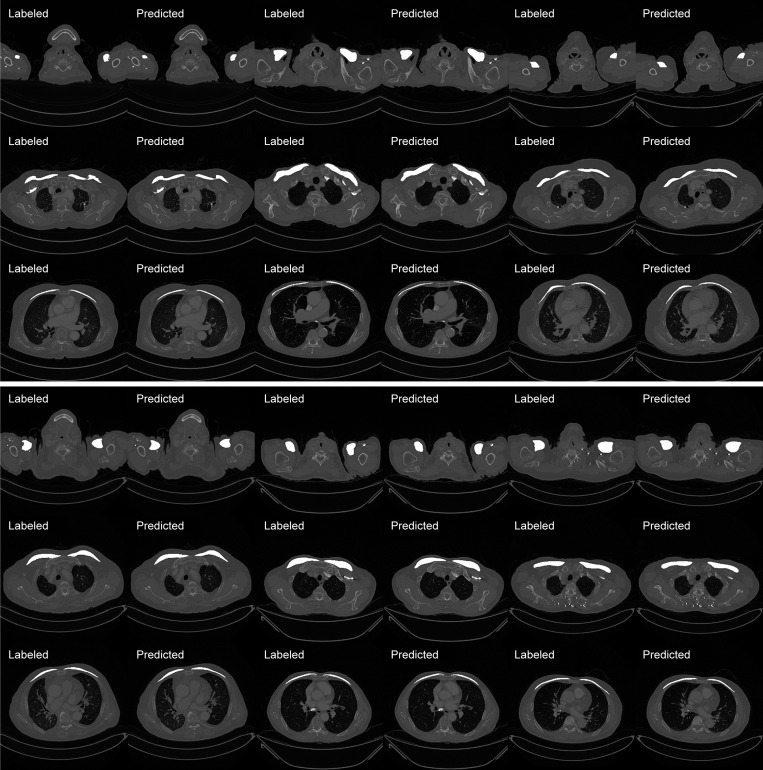
The labeled image of the patient CT dataset and its predicted result using the proposed method.

**Fig 6:**
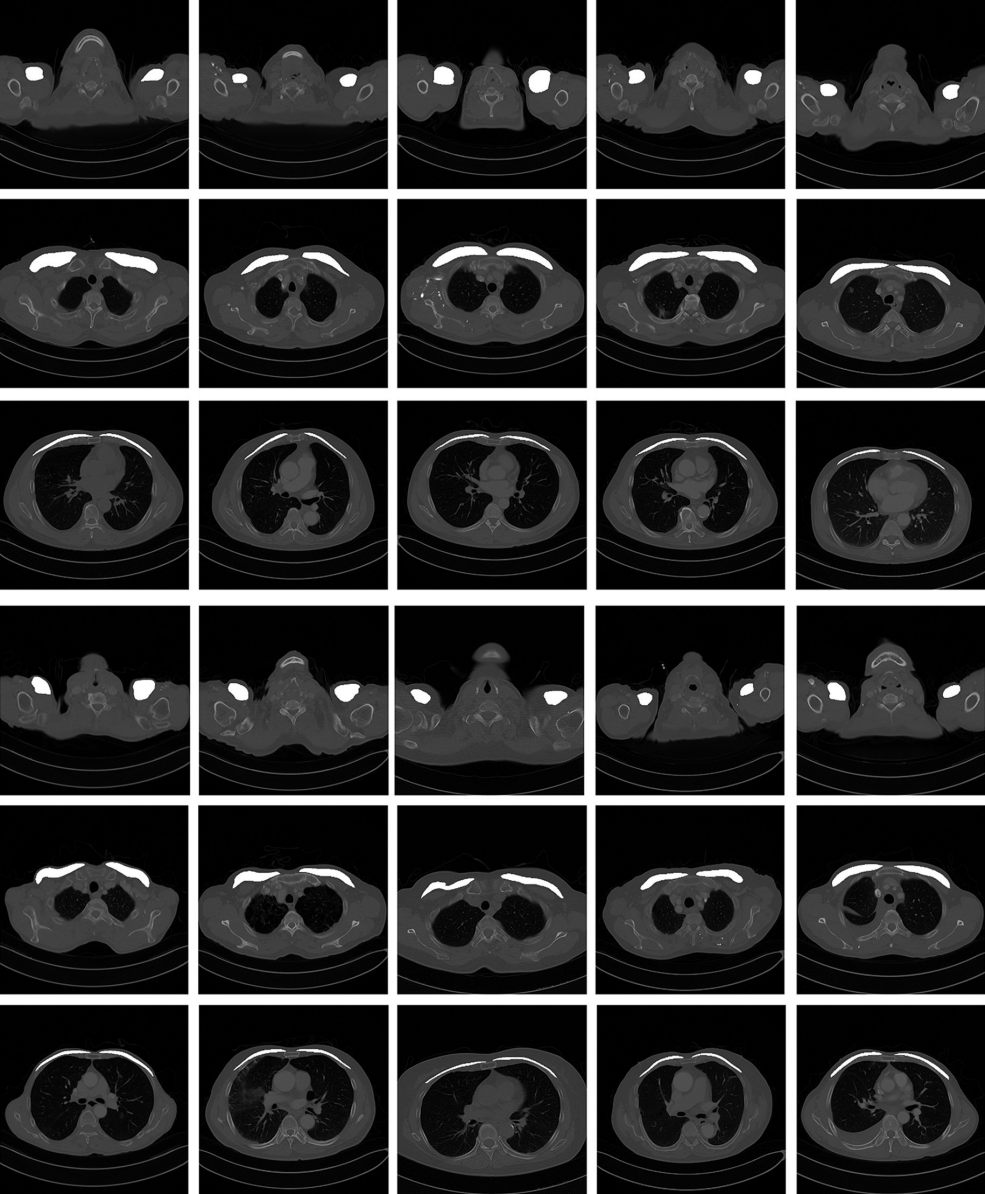
Sample results of the predicted pectoralis muscle area from the patient CT image dataset.

The segmentation results of the proposed method are presented in Figs 5 and 6. The comparison results with the labeled dataset and its predicted result are presented in Fig 5 and the overall volume of the predicted pectoralis muscle is presented in Fig 6.

In the proposed method, the training accuracy and loss value for the pectoralis range detection model were 0.9954 and 0.0725, respectively. For the segmentation model, the loss values were 0.9836 and 0.1725.

Upon visual assessment by experienced radiologists, our method received a score of 9.3 out of 10 points for pectoralis muscle range detection and a score of 8.7 points for overall mask segmentation. The radiologists noted that while the overall performance of the segmentation was good, there were occasional minor issues, such as leakage of the mammary gland tissue. The overall visual assessment results are presented in Table 2.

**Table 2:** The visual assessment score of proposed method in active learning process.

	First dataset	Second dataset	Third dataset	Final dataset
**Range accuracy**	6.6	8.9	9.3	9.3
**Slice segmentation accuracy**	7.1	8.5	9.0	9.2
**Volume segmentation accuracy**	5.4	7.9	8.7	8.7
**Under-segmented**	6.6	3.2	3.5	2.7
**Over-segmented**	1.3	1.1	1.1	1.1

**Fig 7:**
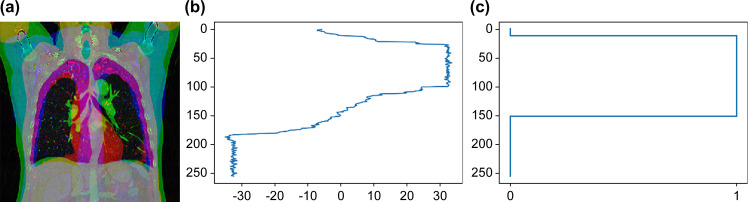
Sample result of the predicted range from the patient CT image data. (a) Multichannel fused coronal image used as input data, providing a comprehensive representation of the pectoralis muscle area. (b) The predicted range results from the range detection model, illustrating the model’s ability to segment the pectoralis muscle area in the input data. (c) Binarized range area, showcasing the final segmentation of the pectoralis muscle area after post-processing steps, such as connected-component detection. CT, computed tomography.

The comparison result from the range detection model is presented in Fig 7.

In the evaluation of the proposed method, the volume and density of the predicted pectoralis muscle from two different CT datasets were compared. The comparison results of the volume showed quite small changes despite the different time and scanning parameters. The average ratios of volume and density had standard deviations of 20.68% and 2.14%, respectively. After excluding outliers identified in the visual assessment process, the standard deviations were reduced to 12.97% and 2.05%, respectively. The calculated average ratios and absolute differences of volume and density are presented in Table 3.

**Table 3:** Validation result comparing the pectoralis muscle area in two different CT image datasets.

	Volume	Density (in VOI)
**Average (Total)**	333.29 ± 121.01 (cc)	42.76 ± 17.47 (HU)
**Average (Pre)**	329.44 ± 124.42 (cc)	41.07 ± 15.44 (HU)
**Average (Post)**	337.13 ± 117.63 (cc)	44.46 ± 19.17 (HU)
**Average Pre/Post ratio**	98.63 ± 20.68 (%)	99.71 ± 2.14 (%)
**Average absolute difference**	40.16 ± 37.94 (cc)	10.97 ± 19.72 (HU)
**Average ratio without outlier**	96.92 ± 12.97 (%)	99.67 ± 2.05 (%)
**Average absolute difference without outlier**	35.95 ± 31.44 (cc)	10.48 ± 19.03 (HU)

CT, computed tomography; VOI, volume of interest

## Discussion

In this study, we attempted to develop a deep-learning-based automated volumetric pectoralis muscle segmentation method able to measure volume and muscle mass for various applications with encouraging results. The segmentation model was divided into two steps because we thought that the CT image should be modified minimally; any filtering or interpolation method would ruin the segmentation result of the image because the values were highly related to the distribution of the Hounsfield unit, which was the derivation of the delicate CT physics. Therefore, we tended to use the CT volume as-is. The range detection of the CT volume was separated into an independent model, and the CT slice images were used as-is to the 2D segmentation model.

The range detection method using multichannel images fused from multi-coronal images was designed because the characteristics of the pectoralis muscle range were shown to be different from other ranges of the dataset; it was denser than the chest area, sparse than the abdominal area, and included specific structures, such as the bronchus and shoulder bone. We thought that the axial/sagittal information should be integrated to obtain the precise range; however, we did not wish to use the entire volume to obtain a simple range of the axial image. Therefore, we designed a multichannel image intended to reduce the image dimension without losing all the structural information of the volume.

We defined the pectoralis major muscle from various studies [14, 19]; the ranges were usually set as the clavicle point as the upper origin, the bicipital groove as insertion, and the disconnected point from the abdominal muscle as the lower bound. However, in this study, the measurement of the muscle was conducted using CT, and the mid-clavicle point tended to be unattached, while the bicipital groove was excluded from the chest CT imaging range.

Interestingly, by having range discrimination, the accuracy of the manual drawing of the range was shown to be relatively lower than the results of the proposed method. It was assumed to be a considerably difficult task, even for a specialist, to annotate the precise area of the muscle range in the CT images.

The efficiency and accuracy of each component can be improved by dividing the process into two proposed models. Therefore, we designed the three-dimensional (3D) U-Net model and the proposed model and compared the initial results, which showed that a large amount of segmentation leakage to the abdomen occurred with the 3D U-Net model. In comparison, the number of occurrences of leakage was relatively small with the proposed model, and we assumed that we enhanced the accuracy of the range detection by implementing a dedicated, separated model and additional data augmentation, which could lead to a more accurate final result.

The model derives segmentation results in the form of a 3D volume; however, the upper and lower bounds of the pectoralis major muscle derived with the model remain debatable, which might be owing to the lack of a fully covered muscle label dataset. However, the results in the 2D slice were very encouraging and accurate, and the area segregation with the pectoralis minor muscle was also suitable. Therefore, the proposed method can be used as a segmentation method for 2D slice images.

For data preprocessing, we used CT denoising software because we believed that the noise from the CT image might affect the calculation of the image feature. Currently, most CT scans are performed using a low-dose scan protocol for the radiation safety principle; ALARA stands for “as low as reasonably achievable,” stating that if receiving that dose has no direct benefit, we should try to avoid it even if it is a small dose. The software provides denoising techniques that can be applied to low-dose CT and shows almost the same level of noise reduction as the iterative reconstruction (IR) technique from major CT vendors. Moreover, because the software does not require raw projection data from the scanning process, a denoising technique can be adopted for retrospective CT data. Owing to these advantages, various studies have used CT images, and validation studies have used software for data preprocessing.

The utilization of pectoralis muscle features as an imaging biomarker holds promise; however, manual measurements of these features in specific slice locations in CT volumes can be prone to inaccuracies due to muscle contraction, hold-breath level, and patient positioning during the scanning process [1, 19, 41–46]. This can lead to distorted results and difficulty in accurately measuring the volume of interest.

The former range detection model was designed with the Inception v3 model; however, it was changed to EfficientNet (B6) because the model showed almost the same or better result, with smaller model size and better convenience in coding, variant input types, and templates. EfficientNet simultaneously considers three scaling factors of the model as follows: depth, width, and resolution, which we explored through various studies and methods. Existing models rarely consider multiple scaling factors simultaneously, and only the degree of depth scaling or width scaling was adjusted. EfficientNet achieves higher accuracy with even fewer parameters through compound scaling, leveraging the model for feature extraction.

For the segmentation model, various models were developed and tested, including U-Net, TransFuse-L, HarDNet-MSEG, and Attention U-Net models; among them, Attention U-Net models were selected since they have the best performance and focus only on specific areas of medical images, which would make the model suitable for the segmentation of this dataset.

We evaluated the segmentation performance along with four key metrics as follows: the mean Dice coefficient, mean intersection over union, precision, and accuracy. The precision and accuracy were both calculated using pixel-wise.

We compared our method with other state-of-the-art methods and typical methods using the same dataset. The proposed segmentation method performed well in segmenting the pectoralis muscle. The mean Dice coefficient, mean intersection over union, precision, and accuracy of the proposed segmentation model were 0.972 and 0.949, respectively, whereas those of TransFuse-L, HarDNet-MSEG, and conventional U-Net were 0.896/0.818, 0.938/0.888, 0.940/0.891, respectively.

In the discussion of the obtained results, it is worth noting the variability introduced by the inclusion of the upper bound of the pectoralis muscle, which is attached to the arm. This region is not typically fully scanned in chest CT, hence, contributes to the variability of the results. The average ratios of volume and density showed a significant standard deviation, particularly when this region was included. Upon excluding outliers during the visual assessment, which did not account for the arm range, a decrease in standard deviation was observed. However, it is postulated that the improved accuracy was not solely due to outlier exclusion, but also likely attributed to the exclusion of the arm-attached region of the pectoralis muscle. Future work may address this variability by improving the segmentation accuracy at the boundaries of the muscle and refining the scanning technique to cover the entire muscle region.

This study has a strict limitation in that the pectoralis major muscle segmentation has a relatively small impact on the image biomarker domain. However, it might have very powerful potential in that the amount of muscle development and loss would be a direct factor associated with sarcopenia, which is currently a major concern and a powerful index in the field of rehabilitation medicine.

## Conclusion

The deep learning-based pectoralis muscle segmentation method developed in this study demonstrated promising results in accurately segmenting the pectoralis muscle in chest CT images. The proposed method has the potential to be employed for measuring the pectoralis muscle volume, mass, and density in various clinical and research settings. By facilitating the quantitative assessment of muscle-related parameters, this technique could contribute to the investigation of muscle-related disorders and the evaluation of patient outcomes, further expanding the method’s potential utility.

## References

[1] Diaz AA, Zhou L, Young TP, McDonald ML, Harmouche R, Ross JC, et al. Chest CT measures of muscle and adipose tissue in COPD: gender-based differences in content and in relationships with blood Biomarkers. Acad Radiol. 2014;21: 1255–1261. doi: 10.1016/j.acra.2014.05.013 25088837 PMC4389286

[2] Yang Z, Jin H, Kim JH. Attenuation profile matching: an accurate and scan parameter-robust measurement method for small airway dimensions in low-dose CT scans. Med Phys. 2018. doi: 10.1002/mp.13074 29969838

[3] Marquis K, Debigaré R, Lacasse Y, Leblanc P, Jobin J, Carrier G, et al. Midthigh muscle cross-sectional area is a better predictor of mortality than body mass index in patients with chronic obstructive pulmonary disease. Am J Respir Crit Care Med. 2002;166: 809–813. doi: 10.1164/rccm.2107031 12231489

[4] Shrikrishna D, Hopkinson NS. Skeletal muscle dysfunction in chronic obstructive pulmonary disease. Respir Med COPD Update. 2009;5: 7–13. doi: 10.1016/j.rmedu.2009.01.002

[5] Hwang YI, Park YB, Yoo KH. Recent trends in the prevalence of chronic obstructive pulmonary disease in Korea. Tuberc Respir Dis (Seoul). 2017;80: 226–229. doi: 10.4046/trd.2017.80.3.226 28747954 PMC5526948

[6] Soler-Cataluña JJ, Sánchez-Sánchez L, Martínez-García MA, Sánchez PR, Salcedo E, Navarro M. Mid-arm muscle area is a better predictor of mortality than body mass index in COPD. Chest. 2005;128: 2108–2115. doi: 10.1378/chest.128.4.2108 16236862

[7] Wouters EFM, Creutzberg EC, Schols AMWJ. Systemic effects in COPD. Chest. 2002;121(5 Supplement): 127S–130S. doi: 10.1378/chest.121.5_suppl.127s 12010840

[8] Andreassen H, Vestbo J. Chronic obstructive pulmonary disease as a systemic disease: an epidemiological perspective. Eur Respir J Suppl. 2003;46: 2s–4s. doi: 10.1183/09031936.03.00000203 14621101

[9] Diaz AA, Martinez CH, Harmouche R, Young TP, McDonald ML, Ross JC, et al. Pectoralis muscle area and mortality in smokers without airflow obstruction. Respir Res. 2018;19: 62. doi: 10.1186/s12931-018-0771-6 29636050 PMC5894181

[10] Jones SE, Maddocks M, Kon SSC, Canavan JL, Nolan CM, Clark AL, et al. Sarcopenia in COPD: prevalence, clinical correlates and response to pulmonary rehabilitation. Thorax. 2015;70: 213–218. doi: 10.1136/thoraxjnl-2014-206440 25561517

[11] Shrikrishna D, Patel M, Tanner RJ, Seymour JM, Connolly BA, Puthucheary ZA, et al. Quadriceps wasting and physical inactivity in patients with COPD. Eur Respir J. 2012;40: 1115–1122. doi: 10.1183/09031936.00170111 22362854

[12] Maltais F, Decramer M, Casaburi R, Barreiro E, Burelle Y, Debigaŕe R, et al. An official American Thoracic Society/European Respiratory Society statement: update on limb muscle dysfunction in chronic obstructive pulmonary disease. Am J Respir Crit Care Med. 2014;189: e15–e62. doi: 10.1164/rccm.201402-0373ST 24787074 PMC4098112

[13] Merola P, Gass R, Dorneles RGP, Gazzana M, Gazzoni F, Hochhegger B, et al. Pectoralis muscle area and skeletal muscle strength in patients with ILD. Eur Respir J. 2016;48: PA5035. doi: 10.1183/13993003.congress-2016.pa5035

[14] Nemec U, Heidinger B, Sokas C, Chu L, Eisenberg RL. Diagnosing sarcopenia on thoracic computed tomography: quantitative assessment of skeletal muscle mass in patients undergoing transcatheter aortic valve replacement. Acad Radiol. 2017;24: 1154–1161. doi: 10.1016/j.acra.2017.02.008 28365235

[15] Go SI, Park MJ, Song HN, Kim HG, Kang MH, Kang JH, et al. A comparison of pectoralis versus lumbar skeletal muscle indices for defining sarcopenia in diffuse large B-cell lymphoma ‐ Two are better than one. Oncotarget. 2017;8: 47007–47019. doi: 10.18632/oncotarget.16552 28388585 PMC5564540

[16] Wang L, Filippatos K, Friman O, Hahn HK. Fully automated segmentation of the pectoralis muscle boundary in breast MR images. Progress in Biomedical Optics and Imaging ‐ Proceedings of SPIE. 7963. doi: 10.1117/12.877645

[17] Kinsey CM, San José Estépar R, van der Velden J, Cole BF, Christiani DC, Washko GR. Lower pectoralis muscle area is associated with a worse overall survival in non-small cell lung cancer. Cancer Epidemiol Biomarkers Prev. 2017;26: 38–43. doi: 10.1158/1055-9965.EPI-15-1067 27197281 PMC5116279

[18] Harmouche R, Ross JC, Washko GR, San José Estépar R, B RH, Ross JC, et al. Pectoralis muscle segmentation on CT images based on Bayesian graph cuts with a subject-tailored atlas. Lecture Notes in Computer Science (including subseries Lecture Notes in Artificial Intelligence and Lecture Notes in Bioinformatics), vol. 8848, Springer Verlag; 2014, p. 34–44. doi: 10.1007/978-3-319-13972-2_4

[19] McDonald MLN, Diaz AA, Ross JC, Estepar RSJ, Zhou L, Regan EA, et al. Quantitative computed tomography measures of pectoralis muscle area and disease severity in chronic obstructive pulmonary disease: a cross-sectional study. Ann Am Thorac Soc. 2014;11: 326–334. doi: 10.1513/AnnalsATS.201307-229OC 24558953 PMC4028743

[20] Bak SH, Kwon SO, Han SS, Kim WJ. Computed tomography-derived area and density of pectoralis muscle associated disease severity and longitudinal changes in chronic obstructive pulmonary disease: a case control study. Respir Res. 2019;20: 226. doi: 10.1186/s12931-019-1191-y 31638996 PMC6805427

[21] Ahn H, Kim DW, Ko Y, Ha J, Shin Y, Lee J, et al. Updated systematic review and meta-analysis on diagnostic issues and the prognostic impact of myosteatosis: A new paradigm beyond sarcopenia. Ageing Res Rev. 2021;70:101398. doi: 10.1016/j.arr.2021.101398 34214642

[22] Landi F, Liperoti R, Fusco D, Mastropaolo S, Quattrociocchi D, Proia A, et al. Sarcopenia and mortality among older nursing home residents. J Am Med Dir Assoc. 2012;13: 121–126. doi: 10.1016/j.jamda.2011.07.004 21856243

[23] Kim J, Park JH, Yim J. Effects of respiratory muscle and endurance training using an individualized training device on pulmonary function and exercise capacity in stroke patients. Med Sci Monit. 2014;20: 2543–2549. doi: 10.12659/MSM.891112 25488849 PMC4266259

[24] Rom O, Kaisari S, Aizenbud D, Reznick AZ. Sarcopenia and smoking: a possible cellular model of cigarette smoke effects on muscle protein breakdown. Ann N Y Acad Sci. 2012;1259: 47–53. doi: 10.1111/j.1749-6632.2012.06532.x 22758636

[25] Crisafulli E, Costi S, Fabbri LM, Clini EM. Respiratory muscles training in COPD patients. Int J Chron Obstruct Pulmon Dis. 2007;2: 19–25. doi: 10.2147/copd.2007.2.1.19 18044062 PMC2692111

[26] Hocaoglu E, Ors S, Yildiz O, Inci E. Correlation of pectoralis muscle volume and density with severity of COVID-19 pneumonia in adults. Acad Radiol. 2021;28: 166–172. doi: 10.1016/j.acra.2020.11.017 33281041 PMC7704063

[27] Mitsiopoulos N, Baumgartner RN, Heymsfield SB, Lyons W, Gallagher D, Ross R. Cadaver validation of skeletal muscle measurement by magnetic resonance imaging and computerized tomography. J Appl Physiol (1985). 1998;85: 115–122. doi: 10.1152/jappl.1998.85.1.115 9655763

[28] Shen W, Punyanitya M, Wang Z, Gallagher D, St-Onge MP, Albu J, et al. Total body skeletal muscle and adipose tissue volumes: estimation from a single abdominal cross-sectional image. J Appl Physiol (1985). 2004;97: 2333–2338. doi: 10.1152/japplphysiol.00744.2004 15310748

[29] Mourtzakis M, Prado CMM, Lieffers JR, Reiman T, McCargar LJ, Baracos VE. A practical and precise approach to quantification of body composition in cancer patients using computed tomography images acquired during routine care. Appl Physiol Nutr Metab. 2008;33: 997–1006. doi: 10.1139/H08-075 18923576

[30] Hiasa Y, Otake Y, Takao M, Ogawa T, Sugano N, Sato Y. Automated muscle segmentation from clinical CT using Bayesian U-Net for personalized musculoskeletal modeling. IEEE Trans Med Imaging. 2020;39: 1030–1040. doi: 10.1109/TMI.2019.2940555 31514128

[31] Wang G, Li W, Zuluaga MA, Pratt R, Patel PA, Aertsen M, et al. Interactive medical image segmentation using deep learning with image-specific fine-tuning. IEEE Trans Med Imaging. 2018;37: 1562–1573. doi: 10.1109/TMI.2018.2791721 29969407 PMC6051485

[32] Çiçek Ö, Abdulkadir A, Lienkamp SS, Brox T, Ronneberger O. 3D. U-net: Learning dense volumetric segmentation from sparse annotation. In: Ourselin S, Joskowicz L, Sabuncu M, Unal G, Wells W, editors. Medical Image Computing and Computer-Assisted Intervention–MICCAI 2016. MICCAI 2016. Lecture Notes in Computer Science, vol 9901. Springer, Cham; 2016. pp. 424–432. doi: 10.1007/978-3-319-46723-8_49

[33] González G, Washko GR, José Estépar RS. Multi-structure segmentation from partially labeled datasets. Application to body composition measurements on CT scans. Lecture Notes in Computer Science (including subseries Lecture Notes in Artificial Intelligence and Lecture Notes in Bioinformatics), vol. 11040 LNCS, Springer Verlag; 2018. pp. 215–224. doi: 10.1007/978-3-030-00946-5_22 32494779 PMC7269188

[34] Dutta IN, Nadeem SA, Comellas AP, Hoffman EA, Saha PK. CT-based segmentation of pectoral muscle using deep learning and association of computed metrics with aging and sex. SPIE-Intl Soc Optical Eng; 2022. doi: 10.1117/12.2613073

[35] Kreher R, Hinnerichs M, Preim B, Saalfeld S, Surov A. Deep-learning-based segmentation of skeletal muscle mass in routine abdominal CT scans. In Vivo (Brooklyn). 2022;36: 1807–1811. doi: 10.21873/invivo.12896 35738592 PMC9301401

[36] Godoy IRB, Silva RP, Rodrigues TC, Skaf AY, de Castro Pochini A, Yamada AF. Automatic MRI segmentation of pectoralis major muscle using deep learning. Sci Rep. 2022;12: 5300. doi: 10.1038/s41598-022-09280-z 35351924 PMC8964724

[37] Tan M, Le QV. Efficientnet: rethinking model scaling for convolutional neural networks. In: International conference on machine learning; 2019. pp. 6105–6114.

[38] Tan M, Le QV. Efficientnetv2: smaller models and faster training. In: International conference on machine learning; 2021. pp. 10096–10106.

[39] Srivastava N, Hinton G, Krizhevsky A, Salakhutdinov R. Dropout: a simple way to prevent neural networks from overfitting. J Mach Learn Res. 2014;15: 1929–1958.

[40] Oktay O, Schlemper J, Folgoc LL, Lee M, Heinrich M, Misawa K, et al. Attention u-net: learning where to look for the pancreas. arXiv preprint arXiv:1804.03999. 2018. doi: 10.48550/arXiv.1804.03999

[41] Ogawa E, Nakano Y, Ohara T, Muro S, Hirai T, Sato S, et al. Body mass index in male patients with COPD: correlation with low attenuation areas on CT. Thorax. 2009;64: 20–25. doi: 10.1136/thx.2008.097543 18852156

[42] Sørensen L, Lo P, Ashraf H, Sporring J, Nielsen M, de Bruijne M. Learning COPD sensitive filters in pulmonary CT. Med image comput assist Interv. 2009;12: 699–706. doi: 10.1007/978-3-642-04271-3_85 20426173

[43] Onoe R, Yamashiro T, Handa H, Azagami S, Matsuoka S, Inoue T, et al. 3D-measurement of tracheobronchial angles on inspiratory and expiratory chest CT in COPD: respiratory changes and correlation with airflow limitation. Int J Chron Obstruct Pulmon Dis. 2018;13: 2399–2407. doi: 10.2147/COPD.S165824 30127602 PMC6089108

[44] Haruna A, Muro S, Nakano Y, Ohara T, Hoshino Y, Ogawa E, et al. CT scan findings of emphysema predict mortality in COPD. Chest. 2010;138: 635–640. doi: 10.1378/chest.09-2836 20382712

[45] Cho YH, Seo JB, Kim N, Lee HJ, Hwang HJ, Kim EY, et al. Comparison of a new integral-based half-band method for CT measurement of peripheral airways in COPD with a conventional full-width half-maximum method using both phantom and clinical CT images. J Comput Assist Tomogr. 2015;39: 428–436. doi: 10.1097/RCT.0000000000000218 25700223

[46] Rozenberg D, Mathur S, Herridge M, Goldstein R, Schmidt H, Chowdhury NA, et al. Thoracic muscle cross-sectional area is associated with hospital length of stay post lung transplantation: a retrospective cohort study. Transpl Int. 2017;30: 713–724. doi: 10.1111/tri.12961 28390073

